# Puerperal Convulsions

**Published:** 1877-03

**Authors:** 


### BUFFALO

### Medical and Surgical Journal.

Vol. XVI. Dec'r, 1876, Jan'y, Feb'y and March, 1877. Nos. 5, 6, 7 & 8.

### Original Communications.

:o:

ART. I.—*Puerperal Convulsions. Cases, with Symptoms and Treatment.* By —— FORDYCE, M. D.

[The following paper, read at the meeting of the Medical Association of Central New York, gave rise to considerable discussion, as to the propriety and safety of employing Veratrum Veride in Puerperal Convulsions. We present the paper, leaving our readers to form their own conclusions. ED.]

I have selected this subject, not because of any claim that it is not well understood, or that any management of it can be presented which has not already some precedent, but because the subject is of the most intense interest to the medical man, as well as to their immediate friends, and of those who may be so afflicted.

The disease and the description of it is, and has been the same for centuries. Certain pathological conditions relating to it are believed to be better understood than formerly, but the visible manifestations are unchangeable.

Although I shall not refer to many authorities for what may be said on this occasion, I have the greatest reverence for the opinions of medical authors upon this subject—simply adding the opinion of Dr. Hodge that "the pathology of puerperal convulsions has never been satisfactorily established to the professional mind." In the following part of the text, pp. 299 and 300, although a number of supposed causes are mentioned, and all, without exception, given as pre-existing to pregnancy, he deduces an argument against inducing or hastening delivery—using this language to the accoucher: "He should postpone, as far as practicable, the occurrence of labor, instead of hastening, prematurely, its advent."

And further on says: "Of course by the code of ethics, such women should not marry—should not become mothers."

To these statements and conclusions we shall take exceptions. Puerperal convulsions occur nearly as frequently in women who have had one or more healthy children, as in primipara when certain conditions are present which occasionally attend gestation.

Albuminuria is the principal and the only pathological condition that for any length of time precedes convulsions. Some escape who have this, but we are *always* apprehensive until we know case safely over. Now, the same condition that produces the convulsions in Albuminuria is also sometimes developed during the progress of protracted labor by mechanical pressure of the end of the uterus upon the bladder, and ureters damming the urine upon the kidneys, also perhaps by pressure upon the kidneys the secretion of urine is suspended, and urea is retained in excess in the blood, producing uremic poisoning and convulsions in part of this class of cases; indeed, a sufficient number to account for all the cases of puerperal convulsions.

I apprehend, of course, that this view of the cause of the disease may be objected to, but having made this a subject of investigation, and having attended a large number of cases, I am fully confirmed in my opinion that uremia is the cause of puerperal convulsions. The proof is stronger, also, if we take into consideration the prodroma that usually precede convulsions in pregnancy. We are consulted for sudden sharp pains in the head, confusion of intellect, generally attended with œdema of extremities, scanty secretion and voiding of urine, and that of dark color with general restlessness, and sometimes coma vigil, urine containing albumen, very little, if any, urine giving, in short, nearly all the symptoms of Bright's disease. Now, if we have an opportunity of treating this class of cases prior to the development of convulsions, no disease of pregnancy is more tractable to the use of remedies. I think we may safely estimate that not over fifty of this class will have convulsions, and without treatment not one is safe. Of the remedies, a carefully selected position in lying, and in every way avoiding pressure on the ureters and kidneys by tight clothing, seem to me most important. Then giving some mild saline laxative frequently, as Rochelle salts or Cream of Tartar, continued until the kidneys are able to resume their functions. If circulation is hurried, control it with Verat Viride so as to reduce frequency of pulse to sixty per minute. Then follow with Digitalis in small repeated doses, and the physician will have few cases of convulsions to treat.

But, unfortunately, from the modesty of ladies in gestation we are not permitted to see or pescribe for them until we are suddenly called to attend them for a convulsion. The character of this kind of convulsion is too well known to require minute description, but to more fully illustrate my plan of treatment, I will give a few cases with symptoms and treatment.

Case I. Mrs. C., æt 20, native, light complexion and hair, slender build and a very delicate lady, blue eyes. Was taken ill on twenty-seventh of May, 1874, at 7½ months, Dr. Pany was called to attend her; on the twentieth she had convulsions, and a Homeopathic physician was called in with the Dr.; I was absent from the place at the time, on my return was called in the afternoon of twenty-eighth, found the lady comatose, and at this time had had nine convulsions; face and extremities œdematous; blood oozing from mouth from injury to tongue; breathing stertorous; chloroform had been administered prior to my visit, but as soon as effects ceased another frightful convulsion had followed; pulse 120; patient insensible, but could swallow small quantities of liquids; advised Verat Viride, ten drops; repeat in thirty minutes in reduced doses until vomiting ensued or pulse was reduced to sixty; convulsions controlled with second dose, and third dose reduced pulse to forty-eight; continue Verat as soon as pulse increases above sixty; on the twenty-ninth, towards night, she became conscious, and by the use of Rochelle salts and Cream of Tartar and Acet. Pot. œdema disappeared; she became quite comfortable, and on the twenty-first of June, twenty-three days after attack was delivered of a still-born child in a stage of great decomposition, without convulsions or any unfavorable symptoms, so far as mother was concerned. This lady's health became good afterwards, and she became pregnant again, and on the twenty-eighth of August, 1876, gave birth to a fine healthy little girl. Prior to confinement, three or four weeks, slight œdema made appearance, and albumen in urine. On use of Cream of Tartar and Rochelle salts it all disappeared before confinement, and by using a few drops of Verat Viride whenever pulse was qnick, she had no further symptoms of convulsions. Is now well.

Case II. Miss H.; Irish girl, æt 18; thick set, fleshy; conception illegitimate, and every effort made on her part for concealment; was taken, as near as I can learn, in night of Oct. 17th, 1875; found by parents in the morning insensible and having a convulsion every fifteen or twenty minutes; Dr. Cummings, of Cayuga, was called to see her in the morning of eighteenth of Oct., and found her in this condition, insensible with no evidence of labor having commenced; he saw her have a good many severe convulsions; tongue bitten through and blood running from the mouth; the Dr. gave chloroform and succeeded in controlling the severity of the convulsions; he sent for me to assist him in the case; on consultation we were satisfied nothing could save the girl's life except prompt delivery, although the mouth of the uterus was not dilated larger than the point of the finger; we gave her chloroform so as to produce complete anesthesia, and proceeded to forcibly dilate the womb and turned and delivered a very large full grown dead child; followed delivery with Verat Vir.; had no more convulsions, and in a week the girl was out of doors as well as usual, except sore tongue. This case had been preceded by scanty secretion of urine, and we were obliged to use catheter next day, only once however. Sensibility restored second day after delivery.

Case III. Mrs. C.; American lady; light complexion and thirty years of age; had one healthy living child some six years old; was a woman of good health generally; about seventh month of gestation began to have scanty secretion of urine and feet somewhat swollen; consulted me in regard to her condition; gave her diuretics and laxatives with relief; about eighth month, she having discontinued these remedies, feeling that the trouble of taking was more than she was willing to submit to, the same symptoms reappeared with confusion of intellect and sudden sharp pains in the head and eyes; I then insisted on her taking the remedies and admonished her husband of my fears of convulsions if she neglected them; she improved again and appeared quite well, but again discontinued remedies about a week before her confinement, which took place May 18th, 1875; was taken with labor pains, as usual, in night before, and also with pains in the head soon after; from this time she did not remember anything that occurred; at 6 P. M. she began to have convulsions while I was absent; on my return found her in a frightful convulsion; controlled them partially with chloroform, and as soon as uterus was dilatable, turned and delivered her of a healthy, living child; she had no more convulsions; kept her under Verat Vir until pulse was fully controlled; she made a good recovery.

Case IV. Mrs. H., of Leadyan; American, of English parents; light complexion, thick set young lady of 20 years; first child; feet had been bloated and urine scanty some four weeks before confinement; no remedies had preceded labor, which had commenced in the evening of October 6th, 1876; I was at Philadelphia at the time, and a Homeopathic physician of some 35 years practice was called; the treatment was purely homeopathic; at about 2 o'clock, morning of the 7th, convulsions commenced and continued every twenty minutes until she had twenty-two; consciousness lost with the first; I was expected to return on morning train at 10 o'clock; on arrival met a messenger as I was stepping off the cars, who had been waiting an hour for train; I learned the particulars and at between 11 and 12 o'clock saw the patient; there was not the first evidence of natural sensibility about her; the same stertorous breathing that attends last stages of apoplexy; tongue bitten through in various places, bloody froth oozing from the mouth.

The old doctor was glad to see me for once in his life; he informed me that he had given all his usual specifics for convulsions without the least appreciable effect; he "feared the potincus were not high enough;" he had only the 200th attenuation, if he had only the 4,000th attenuation, he knew he "could have knocked the convulsions higher than a kite." Upon examination I found head presention and Os-uteri partly dilated, and proceeded at once to deliver with forceps of a very feeble, living child; gave chloroform; the mother continued insensible after delivery; she made no more effort to swallow than a dead person, when liquids were put into her mouth; it was two hours or more before I could get any medicine into the system to influence the convulsions in any manner; she had two convulsions before could get a drop of anything down; then succeeded in getting ten drops Verat Vir swallowed; in thirty minutes gave another dose; after the second dose convulsions ceased; she remained unconscious for forty-six hours, when upon hearing the child cry she roused up and asked "what is that?" From this time she became gradually conscious until she was fully restored; the urine in this case was very scanty and removed with catheter twice after delivery; contained albumen and no urea; by using Acet. Potash, the normal quantity and quality of the secretion was restored in two days more; the bloating disappeared and secretion of milk came on so that the child received full nourishment from the mother; recovery good.

These cases are cited to illustrate the effects of the Verat Viride upon convulsions of this character. I have not used it in uremic convulsions from Bright's disease, but I have no doubt of its value in them. It is of great service in convulsions after Scarlet Fever with dropsical sequelæ, which, no doubt depend upon the same cause. Cazeaux and Simpson mention the diseases in the same light.

